# Evaluation of an acrylic acid hydrogel dosimeter for 3-D dose verification in radiotherapy using MRI

**DOI:** 10.1371/journal.pone.0349404

**Published:** 2026-05-14

**Authors:** Ihssan S. Masad, Khalid A. Rabaeh, Samer I. Awad, Akram A. Almousa, Ahmed M. Masawi, M. Abdullah Al Kafi, Samer M. Alheet, Abdallah J. Nofal, Belal Moftah

**Affiliations:** 1 Department of Electrical and Computer Engineering and GUST Engineering & Applied Innovation Research Centre (GEAR), Gulf University for Science and Technology (GUST), Hawally, Kuwait; 2 Department of Biomedical Systems and Informatics Engineering, Hijjawi Faculty for Engineering Technology, Yarmouk University, Irbid, Jordan; 3 Medical Imaging Department, Faculty of Applied Medical Sciences, The Hashemite University, Zarqa, Jordan; 4 Department of Biomedical Engineering, Faculty of Engineering, The Hashemite University, Zarqa, Jordan; 5 Biomedical Physics Department, Research Center, King Faisal Specialist Hospital and Research Center, Riyadh, Saudi Arabia; 6 Radiotherapy Department, King Hussein Cancer Center, Amman, Jordan; 7 Diagnostic Radiology Department, King Hussein Cancer Center, Amman, Jordan; 8 Medical and Clinical Affairs, King Faisal Specialist Hospital and Research Centre, Madinah, Saudi Arabia; 9 Medical Physics Unit, McGill University, Montréal, Québec, Canada; Radiation Application Research School, NSTRI, IRAN, ISLAMIC REPUBLIC OF

## Abstract

Gel dosimeters are commonly used to verify the dose distribution and consequently, ensure the accurate delivery of the dose as planned, which is very critical for the success of the radiotherapy. The current study aims to investigate the feasibility of using the magnetic resonance imaging (MRI)-based relaxation rate maps (R2 maps) of the novel acrylic acid with organic glucose polyvinyl alcohol (ACAGLPVA) hydrogel dosimeter for 3-D dose verification. The (ACAGLPVA) hydrogel was prepared and scanned with a 3-T MRI, prior to irradiation, using a spin-echo (SE) sequence. A series of 30 images with different echo times (TE) were acquired in order to generate R2 maps. The hydrogel was then irradiated with a maximum dose of 8 Gy using a CyberKnife system and the same MRI scans were acquired again for the irradiated gel phantom. The measured dose distributions obtained from R2-MRI maps were compared, using 2-D and 3-D Gamma analyses with 3% dose difference and 3-mm distance-to-agreement criteria, to those calculated from the treatment planning system (TPS). Results have shown high spatial agreement between the dose distributions calculated from TPS and the dose distributions derived from R2 maps, particularly in high-dose regions. The 3-D Gamma analysis achieved pass rates exceeding 90% across all planes for all evaluated dose levels—namely 90%, 70%, 50% and 20% of the maximum dose in the axial plane, as well as 90%, 70% and 50% of the maximum dose in the coronal and sagittal planes. In conclusion, based on the analysis of R2-MRI maps, this study has demonstrated the reliability of using the novel (ACAGLPVA) hydrogel dosimeter as an accurate tool for 3-D verification of radiotherapy treatment plans.

## 1. Introduction

The accurate delivery of radiation dose as planned is essential for the success of radiotherapy, particularly with modern treatment techniques that demand high spatial precision, such as total body irradiation (TBI) [1], intensity modulated radiotherapy (IMRT) [2], volumetric modulated arc therapy (VMAT) [3] and stereotactic body radiotherapy (SBRT) [4]. These advanced techniques involve complex beam arrangements and steep dose gradients, making reliable patient-specific quality assurance (PSQA) a critical component of clinical practice. Polymer gel dosimeters have attracted significant interest for radiotherapy verification because of their near tissue-equivalent properties and their ability to record absorbed dose distributions in three dimensions [5,6]. Unlike conventional dosimetry tools such as ionization chambers, thermoluminescent dosimeters (TLDs) and film dosimetry—which are inherently limited to point or planar measurements—polymer gel dosimeters enable true volumetric dose verification [7–9]. While early gel formulations raised concerns related to toxicity and handling, more recent polymer gel compositions have been developed to improve safety, mechanical stability and radiation sensitivity [10–12].

Radiation exposure induces chemical and physical changes in polymer gels that can be correlated with absorbed dose. These changes may manifest in optical properties or magnetic properties, which can be quantified using appropriate readout techniques. One-dimensional dose–response characterization has been widely performed using ultraviolet–visible (UV–vis) spectrophotometry to assess radiation-induced optical changes [13–16], as well as nuclear magnetic resonance (NMR) methods to quantify variations in longitudinal and transverse relaxation parameters [10,17]. Although these approaches provide valuable dosimetric information, they do not fully exploit the intrinsic three-dimensional recording capability of gel dosimeters.

To enable spatially resolved dose verification in two and three dimensions, several imaging modalities have been employed, including ultrasound [18,19], computed tomography (CT) [20–22], optical computed tomography (OCT) [12,23,24] and magnetic resonance imaging (MRI) [25–28]. In particular, CT- and cone-beam CT–based readout of polymer gels has gained renewed attention due to its widespread clinical availability. Recent work by Lumley et al. [29] demonstrated the development of a high-sensitivity PASSAG-based polymer gel optimized for X-ray CT readout, achieving improved dose sensitivity and linear response over clinically relevant dose ranges. Similarly, Kozicki et al. [30] reported the use of multiple polymer gel formulations combined with iterative cone-beam CT and dedicated 3D analysis software for routine radiotherapy quality assurance, highlighting the growing clinical interest in CT-based volumetric gel dosimetry systems.

MRI-based readout remains an attractive alternative for polymer gel dosimetry due to its high soft-tissue contrast, absence of ionizing radiation during readout and ability to generate voxel-wise maps of intrinsic magnetic parameters such as T1, T2, R1 and R2. MRI-derived relaxation rate mapping enables radiation-induced changes in gel properties to be directly correlated with absorbed dose distributions in two and three dimensions [25–28]. However, the dosimetric performance of MRI-based gel systems remains highly dependent on gel formulation, imaging protocol and post-processing methodology, particularly for small-field and stereotactic applications.

Among various polymer gel formulations, acrylic acid (ACA)–based hydrogels incorporated within a polyvinyl alcohol (PVA) matrix have demonstrated favorable dose sensitivity and mechanical stability. Previous studies on ACA-based gels sensitized with inorganic salts, such as MgCl₂, have evaluated radiation-induced optical changes using UV–vis spectrophotometry and OCT, showing linear dose response and suitability for three-dimensional radiotherapy verification [12,31]. More recently, the magnetic response of ACA-based hydrogel dosimeters has been investigated using NMR techniques, demonstrating a strong linear relationship between absorbed dose and the transverse relaxation rate (R2) in one-dimensional measurements [32].

Despite these promising findings, the application of MRI-derived R2 mapping for two- and three-dimensional dose verification using ACA-based hydrogels has not yet been comprehensively evaluated, particularly in the context of stereotactic radiosurgery systems such as CyberKnife, where small field sizes and steep dose gradients impose stringent dosimetric requirements. Therefore, the aim of this study is to evaluate the feasibility and accuracy of using MRI-based R2 maps of an acrylic acid hydrogel dosimeter for three-dimensional verification of radiotherapy treatment plans. In this work, the ACA–PVA gel formulation is further modified by employing organic glucose as a sensitizer instead of inorganic MgCl₂ to enhance formulation originality. The proposed approach is assessed through volumetric comparison of measured and treatment planning system–calculated dose distributions using two- and three-dimensional gamma analysis for a CyberKnife patient-specific quality assurance plan.

## 2. Materials and methods

### 2.1. Gel dosimeter preparation

An acrylic acid with glucose polyvinyl alcohol (ACAGLPVA) polymer gel dosimeter was fabricated in fume hood under normal conditions. The utilized chemicals, purchased from Aldrich in Germany, included acrylic acid (ACA), N,N-methylene-bis-acrylamide (BIS), polyvinyl alcohol (PVA), D-(+)-glucose (GL), glutaraldehyde (GTA) and tetrakis (hydroxymethyl) phosphonium chloride (THPC). An aqueous solution of GL was initially prepared by combining 370 g of GL powder with 900 g of triple distilled water at room temperature, followed by stirring for approximately 10 minutes to achieve a clear solution. Following the elevation of the GL solution temperature to 80 °C, 70 g of PVA was incorporated and the mixture was stirred for approximately two hours. Subsequently, the temperature of the homogeneous GL-PVA solution was lowered to 50 °C to introduce 42 g of BIS (over a duration of about 60 minutes), 7 g of ACA (approximately 5 minutes), 7 g of GTA (around 3 minutes) and 4 g of THPC (also about 3 minutes), respectively. The final polymeric PVA gel (ACAGLPVA) was cooled to about 35 °C and subsequently transferred into an airtight large glass bottle (about 950 mm) and a small glass tubes (about 25 mm). After preparation, the gel phantoms were sealed in airtight glass containers and stored at room temperature away from direct light to minimize environmental effects. MRI scanning prior to irradiation was performed after the gel had reached thermal equilibrium with the scanner room. Following irradiation, the gel phantom was stored under identical conditions and MRI acquisition was performed within a consistent time window to reduce temporal and temperature-related variability in R2 measurements.

### 2.2. Irradiation

The patient-specific quality assurance (PSQA) serves as a comprehensive end-to-end verification of the entire treatment workflow. This process validates both the plan’s integrity and delivery precision as well as a critical safeguard to detect any discrepancies that might arise when transferring the calculated plan into physical irradiation. In this study, the PSQA and analysis were conducted in accordance with the recommendations outlined in the American Association of Physicists in Medicine Task Group 135 (AAPM TG-135) report [33].

The CyberKnife (CK) radiotherapy machine (Accuray Inc., Sunnyvale, CA, USA) uses a nominal 6 MV flattening filter free (FFF) beam. The studied plan used 111 non-isocentric and non-coplanar beams for a brain metastesis patient. The 111 beams used in this study were delivered from multiple “nodes” (points in space where the robotic arm stops to deliver a beam) of the CK system. This high number of beams is characteristic of CK’s ability to create highly conformal dose distributions with a steep dose gradient, which is essential for sparing healthy brain tissue surrounding a metastasis. The treatment plan utilized 5 mm and 7.5 mm Fixed Cones to balance treatment efficiency with dose conformity. These specific sizes were selected based on the target shape and volume of the brain metastasis in this study. Typically, the smaller cones are used to sharpen the dose gradient at the edges of the target or to treat small outcroppings, while relatively larger cones are used to efficiently deliver the dose to the “core” of the lesion. Since fixed collimators are used here (not the Iris variable aperture or Multileaf Collimator), the beam diameter is constant for all beams assigned to that specific cone size.

**Fig 1:**
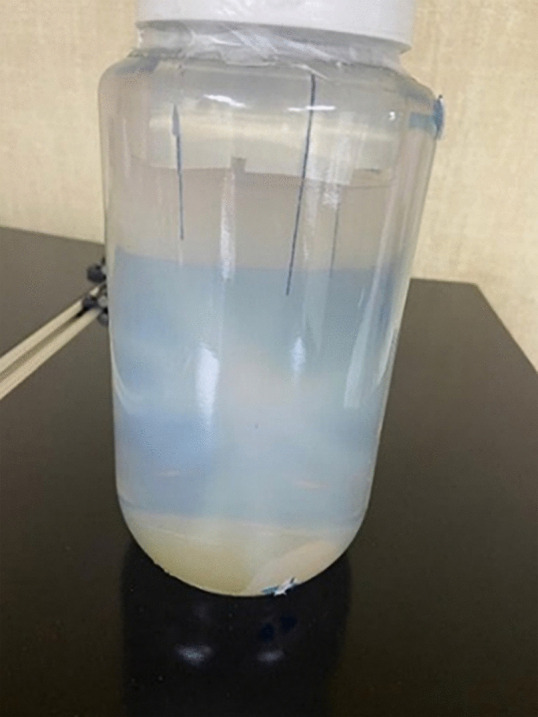
An off-center titanium wire in the hydrogel container.

**Fig 2:**
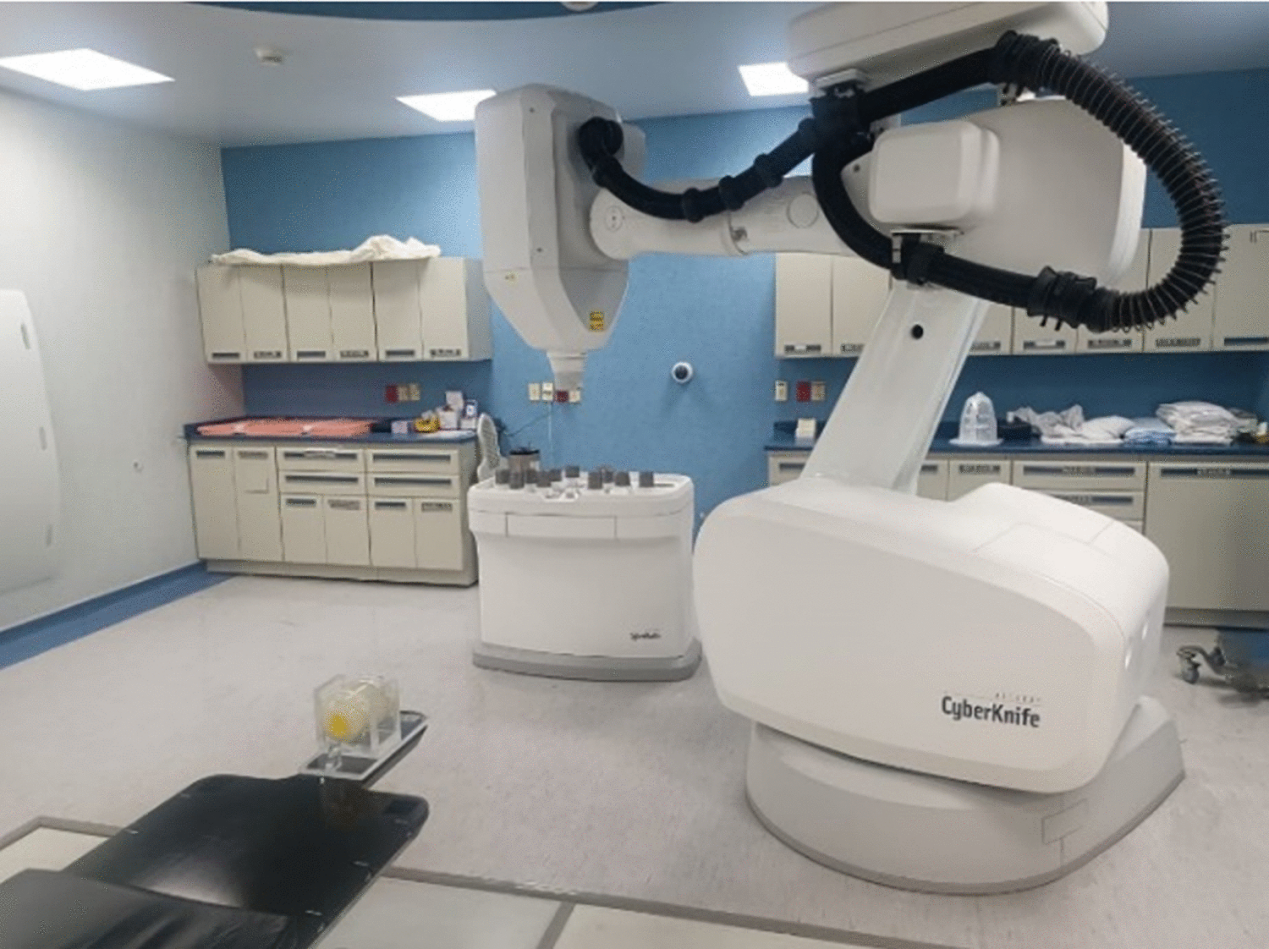
The glass phantom gel sample mounted using a special acrylic holder in the CyberKnife system (Accuray, Sunnyvale, CA).

The CT scan of the 3D gel phantom was used to mimic the patient’s anatomy. The 3D CT image volume of the 3D gel phantom was acquired using a CT scanner (Brilliance Big Bore, Philips Radiation Oncology Systems, Fitchburg, WI). The patient’s clinical dose distribution (planned dose) was mapped onto this phantom geometry within the CyberKnife treatment planning system (TPS) (Multiplan, version 5.1, Accuray Inc., Sunnyvale, CA, USA) to generate a PSQA plan. The resulting PSQA plan DICOM image used for this study had the size of 185 × 185 × 160 voxels with a resolution of 0.68 × 0.68 × 1 mm^3^. The hydrogel phantom was designed with an acrylic holder and gold fiducial markers that are placed at various locations to facilitate fiducial tracking during irradiation. Additionally, an off-center titanium wire, measuring 0.4 mm in diameter, was embedded in the hydrogel container (Fig 1) for image registration across the CT scan, TPS plan, and MRI scan. The PSQA dose plan was transferred to the CyberKnife robotic SRS/SBRT system for irradiation (Fig 2). During irradiation, the CK imaging system identifies the gold fiducial markers embedded in the phantom by utilizing the fiducial tracking method. This allows the robot to compensate for any slight setup misalignments in real-time, mimicking the tracking used during an actual brain metastasis treatment. While the original clinical calculated plan was 21 Gy, the PSQA plan was proportionally rescaled to a maximum dose of 8 Gy for the PSQA delivery. This adjustment was done so that the irradiated dose remained within the dosimeter’s optimal response range while maintaining the original delivery geometry, including the 111-beams and the 5 mm and 7.5 mm cones.

### 2.3. Magnetic resonance imaging (MRI)

**Fig 3:**
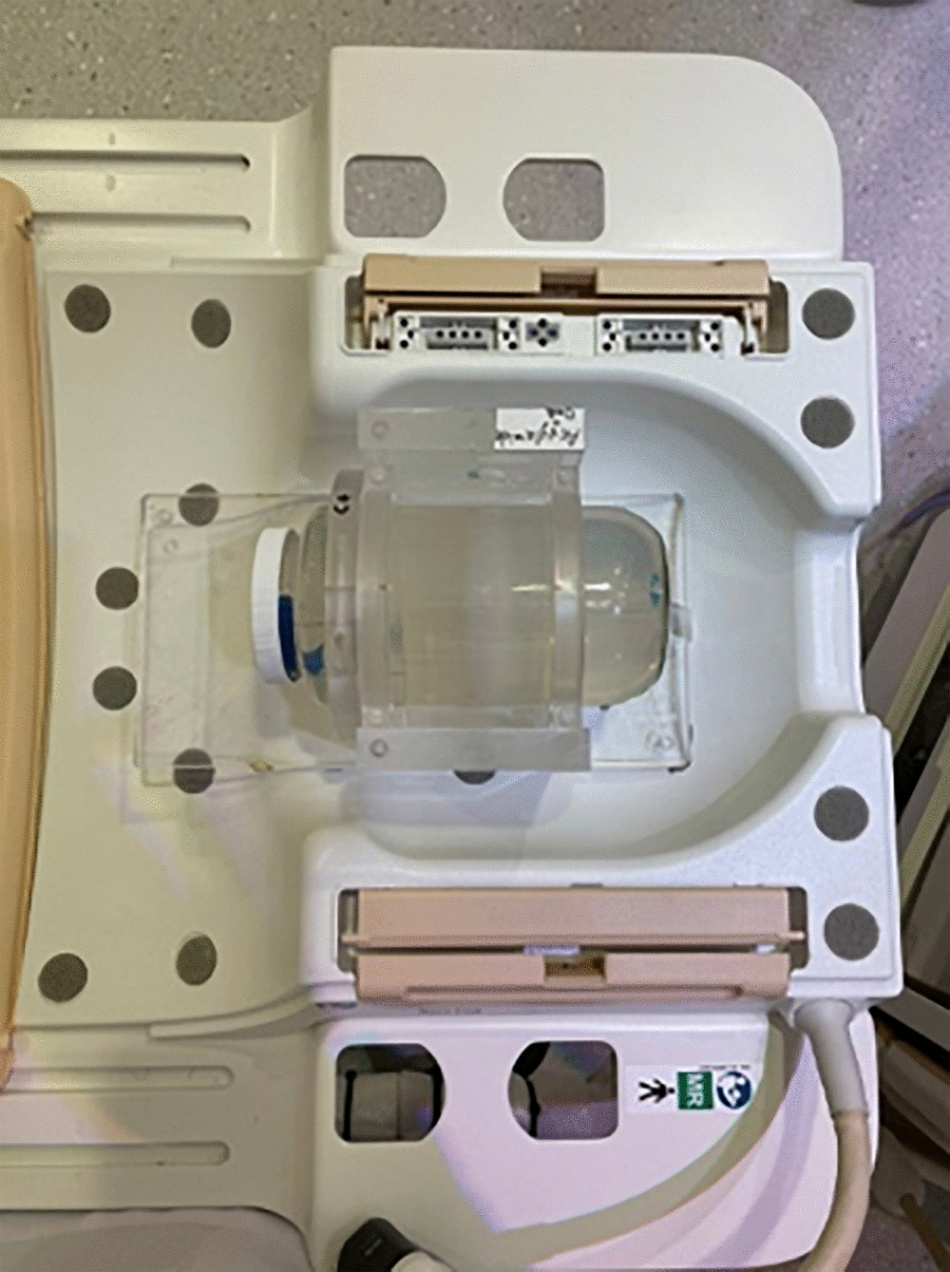
Photograph of the ACAGLPVA gel phantom mounted in a custom acrylic holder and positioned inside the MRI head coil. The setup illustrates the phantom geometry and imaging configuration used for pre- and post-irradiation MRI acquisition.

Pre-and post-irradiation MRI scans of the (ACAGLPVA) gel phantom were acquired using a whole-body 3-T MRI scanner (Philips Medical Systems, USA). The gel phantom was positioned centrally within the MRI head coil using a custom acrylic holder to ensure reproducible orientation and minimize motion during scanning. Alignment was performed utilizing the built-in orthogonal laser beams in the scanner such that the principal axes of the phantom were approximately parallel to the scanner coordinate system. The same positioning and setup were used for CT scans, as well as for pre- and post-irradiation MRI acquisitions to ensure spatial consistency between datasets and minimize the required registration between images. Fig 3 illustrates the prepared gel phantom mounted in its acrylic holder and positioned within the MRI head coil, providing a visual reference for the phantom geometry and imaging setup used throughout the study.

A **32-** channels head coil was utilized to transmit/receive signal. All MR images were acquired using a classic Spin-Echo (SE) sequence with 1-mm slice thickness, the reconstructed field of view (FOV) in the image plane was 230 mm × 230 mm and the matrix size was 230 × 230, resulting in an isotropic in-plane resolution of (1 mm^2^).

In order to generate R2 maps, a series of 30 MR images were acquired using a spin-echo sequence with echo times (TE) ranging from 40 ms to 1200 ms in 40 ms increments, while the repetition time (TR) was kept constant at 7000 ms. The spin-echo sequence was selected due to its robustness and widespread use in polymer gel dosimetry, providing reliable mono-exponential signal decay behavior for R2 estimation. The long TR was used to minimize T1-weighting effects, whereas the wide TE range ensured adequate sampling of signal decay for accurate voxel-wise R2 fitting. These parameters were chosen to balance signal-to-noise ratio, fitting stability and total acquisition time. R2 maps were generated using Parametric Magnetic Resonance Imaging v1.3.3-b (pMRI) software (https://www.parametricmri.com), developed by The Children’s Hospital of Philadelphia (Philadelphia, PA, USA). The generation of the R2 maps was based on the exponential fit of the SE pulse sequence steady state equation, assuming TE to be much shorter than TR (TE ≪ TR) as shown in eq (1) below:


$$\begin{array}{c} S=S_{\text{0}}e^{-(R\text{2}\times TE)}+C \end{array}$$ (1)

Where S is the signal intensity,$\;S_{\text{0}}$ is the initial signal intensity, R2 is the transverse relaxation rate, TE is the echo times and C is constant.

An independent experiment was conducted to create the R2/Dose calibration curve for the (ACAGLPVA) gel. In this experiment, a phantom made of the (ACAGLPVA) gel was exposed to three doses by the CyberKnife system (4, 8 and 12 Gy) at three different locations in the phantom, making spheres of 7-mm diameter. The locations of the three doses were kept apart by at least a distance of 50 mm to minimize the effect of radiation scattering.

The MRI acquisition parameters were identical to those described above and R2 maps were generated using the pMRI software. Regions of interest (ROIs) were selected within the central, high-dose plateau of each irradiated sphere to minimize partial-volume effects and the influence of steep dose gradients and the average R2 values within these ROIs were calculated. In addition, a fourth ROI was selected in a no-dose region and its average R2 value was determined. The no-dose R2 value was independently confirmed by analyzing a corresponding ROI in the pre-irradiation MRI images of the gel phantom.

**Fig 4:**
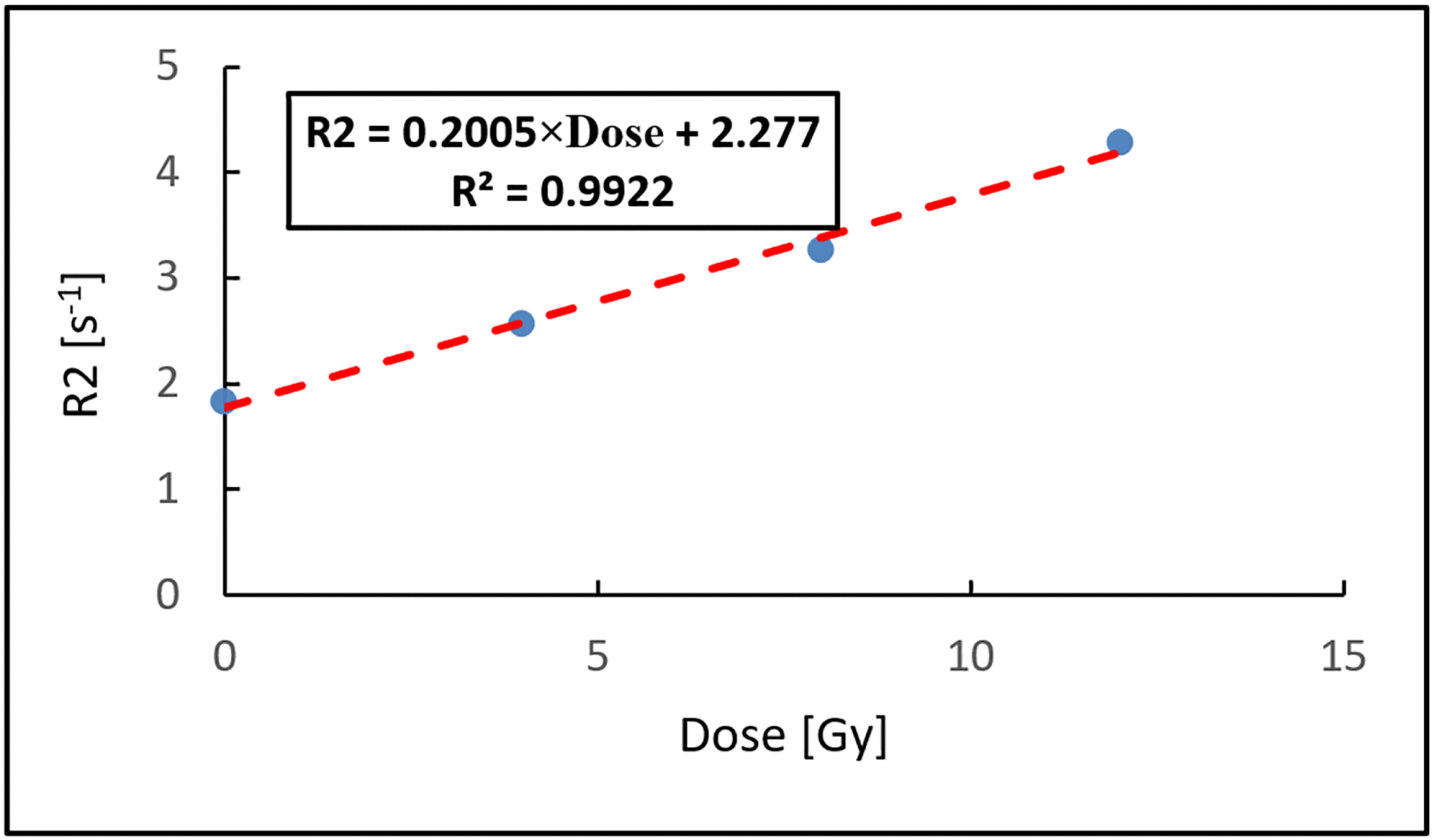
The R2–dose calibration curve for the ACAGLPVA hydrogel dosimeter. The plot shows the average R2 values measured within the selected regions of interest (ROIs) as a function of the delivered dose. The red dashed line represents the linear regression fit used to establish the R2-to-dose calibration relationship.

Fig 4 shows the resulting R2–dose calibration curve, constructed from the average R2 values of the irradiated and no-dose ROIs plotted against their corresponding dose values. Linear regression was used to determine the calibration relationship, yielding a standard error of estimate (SEE) of 0.112 and a coefficient of determination (R^2^) of 0.9922, indicating a strong linear correlation between R2 and absorbed dose. Image noise was mitigated through voxel averaging within each ROI, while uncertainty in R2 estimation primarily arises from signal noise and mono-exponential fitting error. A comprehensive uncertainty propagation and repeatability analysis was beyond the scope of this feasibility study and will be addressed in future.

### 2.4. 2-D and 3-D gamma analysis

After the completion of the registration and resolution matching between the TPS and R2-MRI maps, the measured dose distribution obtained from R2-MRI data was compared to the planned dose distribution obtained from the TPS using Gamma analysis. Gamma analysis quantitatively evaluated the agreement between the planned and measured dose distributions in terms of dosimetric performance, represented by the dose percent error and in terms of spatial accuracy, represented by the distance-to-agreement (DTA) [34,35]. 2-D Gamma analysis of the central axial slice as well as 3D Gamma analysis were performed using an in-house developed MATLAB code (MathWorks Inc., Natick, MA, USA). The passing criteria were 3% dose difference and 3-mm DTA.

The gamma evaluation criterion of 3% dose difference and 3 mm distance-to-agreement was selected in accordance with widely adopted clinical patient-specific quality assurance practices and recommendations for end-to-end verification in stereotactic radiotherapy systems (e.g., AAPM TG-135 and TG-218). While tighter criteria such as 2%/2 mm or 1%/1 mm are often applied in small-field dosimetry studies, the present work aims to establish the feasibility of MRI-based R2 mapping for volumetric gel dosimetry under clinically representative conditions. The selected criterion therefore provides a balanced assessment of both dosimetric and spatial agreement while accounting for uncertainties related to gel formulation, MRI voxel size and multi-modality image registration.

## 3. Results and discussion

### 3.1. Dose distributions and isodose curves

**Fig 5:**
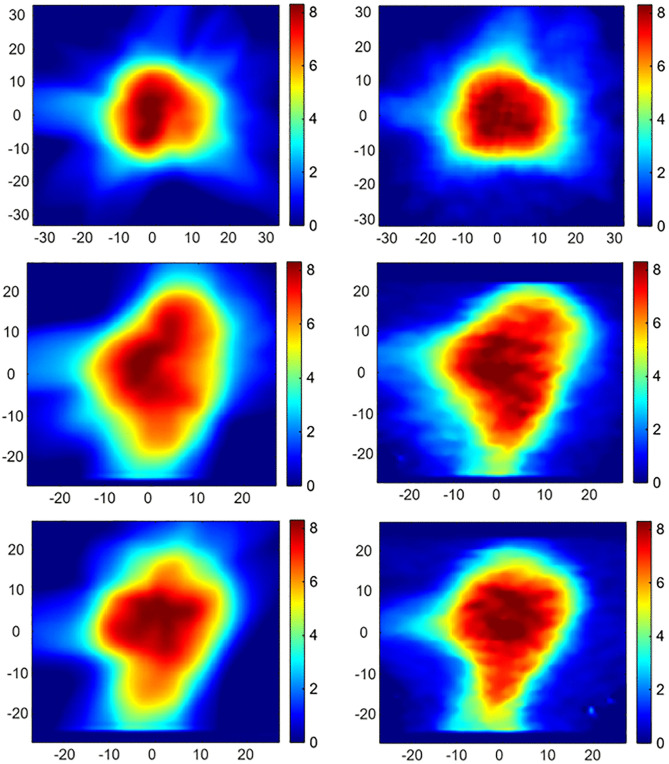
Spatial dose distributions obtained from the treatment planning system (TPS) and the MRI-derived R2 maps of the ACAGLPVA gel dosimeter. The left and right columns show the TPS-calculated and R2-MRI–derived dose distributions, respectively. The top, middle and bottom rows correspond to the central axial, coronal and sagittal slices, respectively. The color scale represents dose values ranging from 0 to 8 Gy.

Fig 5 compares the dose distributions obtained from the TPS (left column in Fig 5) to those derived from the R2-MRI maps (right column in Fig 5) of the irradiated (ACAGLPVA) gel dosimeter in the central slices of the axial, coronal and sagittal planes (top, middle and bottom rows of Fig 5, respectively).

Fig 5 shows the spatial dose variations within the gel phantom illustrated by the color scale that ranges from blue (0 Gy) to red (8 Gy). Overall, both TPS and R2-MRI maps demonstrate similar spatial patterns of dose distributions. However, minor differences were observed, particularly in the sagittal and coronal cross sections. In addition, the limited number of slices acquired in MRI resulted in a limited field of view in the depth direction.

**Fig 6:**
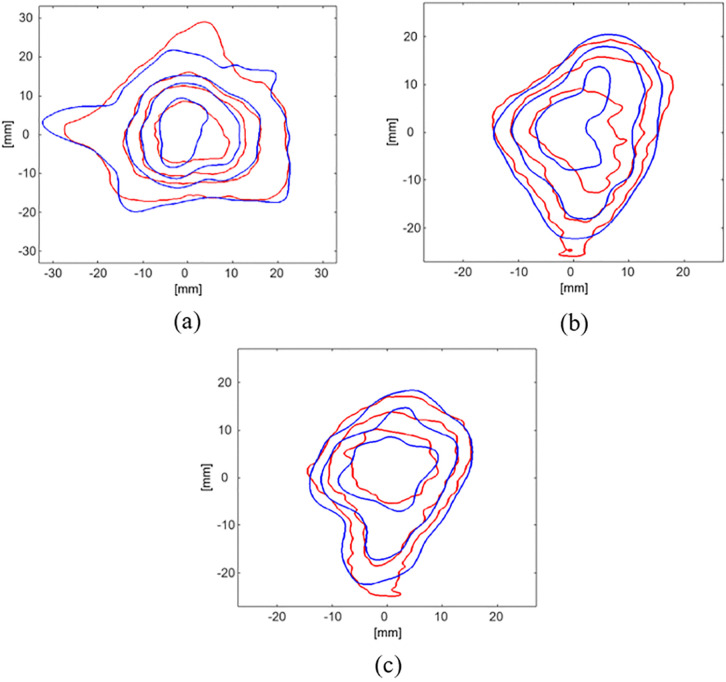
Isodose contours derived from the treatment planning system (TPS) and the MRI-based R2 dose maps of the ACAGLPVA gel dosimeter. Blue and red contours represent the TPS-calculated and R2-MRI–derived dose distributions, respectively. Panels **(a)**, (b) and (c) correspond to the central axial, coronal and sagittal slices. From the innermost to the outermost contours, the dose levels represent 90%, 70%, 50% and 20% of the maximum dose (8 Gy). The 20% isodose contour is not shown in the coronal and sagittal views due to the limited MRI field of view, as discussed in the text.

On the other hand, Fig 6 shows the isodose curves of the dose distribution obtained from TPS (Blue curves) and R2-MRI maps (Red curves). The contours in the axial slice (Fig 6-a) correspond to dose levels of 90%, 70%, 50% and 20% of the maximum dose of 8 Gy from the inner layer to the outer layer, respectively; while contours in the coronal and sagittal central slices (Fig 6-b and 6-c, respectively) correspond to dose levels of 90%, 70% and 50% of the maximum dose. As mentioned above, the FOV was limited in the depth direction and accordingly, the contour of the 20% of maximum dose was excluded from the coronal and sagittal isodose curves due to the lack of acquired MRI data.

The close alignment between the blue (TPS) and red (R2-MRI maps) curves indicates a high agreement between the planned and measured dose distributions. However, minor misalignments between the curves can be observed, particularly in the axial and coronal slices. These deviations may be attributed to the limitation of MRI spatial resolution or some errors in the registration process between the TPS and MRI datasets.

### 3.2. 2-D gamma analysis

**Fig 7:**
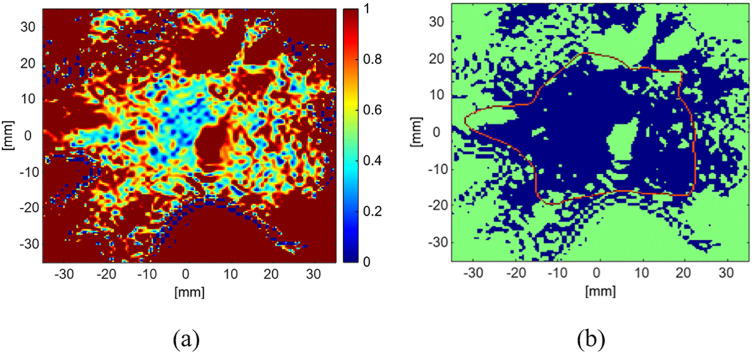
Two-dimensional gamma analysis comparing the TPS-calculated dose distribution and the R2-MRI–derived dose distribution in the central axial slice using a gamma criterion of 3%/3 mm. Panel (a) shows the gamma index map, while panel (b) shows the corresponding satisfactory agreement map, where passing pixels (γ < 1) are displayed in blue. The red contour in panel (b) represents the 20% isodose line of the TPS-calculated maximum dose (8 Gy).

2-D Gamma analysis was performed on the axial central slice in order to evaluate the agreement between the TPS dose distribution and the R2-MRI dose distribution. Results of the 2-D Gamma analysis are illustrated by the gamma index map that is shown in Fig 7-a using 3% dose difference and 3 mm DTA criteria. Pixels with gamma index values less than one indicate satisfactory agreement (Blue area in Fig 7-b). The red contour in Fig 7-b delineates the 20% of the TPS-calculated maximum dose isodose line.

It was observed that the majority of the high dose region showed gamma index values less than one indicating good dosimetric agreement. However, in order to quantitatively evaluate the dosimetric agreement, the achieved pass rates were calculated within regions determined by the isodose lines of different percentages of the TPS-calculated maximum dose of 8 Gy as shown in Table 1. The results suggested high agreement between the TPS-calculated and R2-MRI measured dose distributions, especially in the high dose regions.

**Table 1:** 2-D Gamma analysis (3%/ 3 mm) pass rates calculated within regions at different percentages of the TPS-calculated maximum dose of 8 Gy.

Percentage of max. dose in TPS data	Pass rate
90%	98.9%
70%	85.4%
50%	87.8%
20%	81.3%

### 3.3. 3-D gamma analysis

**Fig 8:**
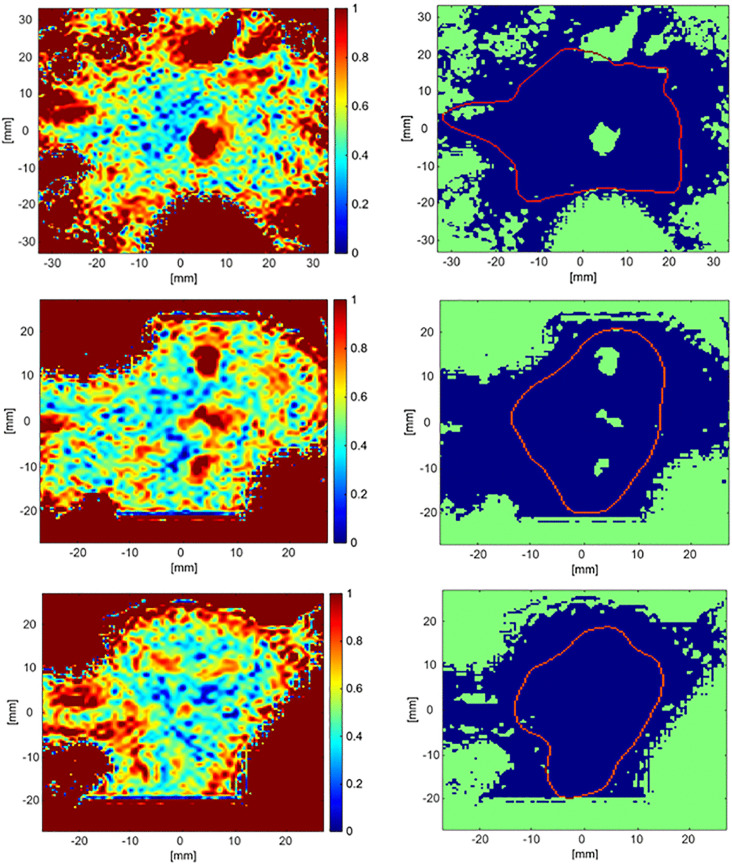
Three-dimensional gamma analysis comparing the TPS-calculated and R2-MRI–derived dose distributions using a gamma criterion of 3%/3 mm. The top, middle and bottom rows correspond to the central axial, coronal and sagittal slices, respectively. The left column displays the gamma index maps, while the right column shows the corresponding satisfactory agreement maps, with passing pixels (γ < 1) indicated in blue. In the satisfactory agreement maps, the red contour represents the 20% isodose line of the maximum dose (8 Gy) in the axial slice and the 50% isodose line in the coronal and sagittal slices. The 20% isodose contour is not shown in the coronal and sagittal views due to the limited MRI field of view, as discussed in the text.

3-D Gamma analysis using 3% dose difference and 3 mm DTA criteria was performed for the TPS and the R2-MRI dose distributions. The 3-D gamma index maps as well as the satisfactory agreement maps were generated for the axial, coronal and sagittal central slices as shown in Fig 8 (top, middle and bottom rows, respectively). The red contour in the axial satisfactory agreement map delineates the 20% of the TPS-calculated maximum dose isodose line, while it delineates the 50% of the TPS-calculated maximum dose isodose line in the coronal and sagittal satisfactory agreement maps. The 20% of the maximum dose was excluded in the coronal and sagittal views due to the lack of acquired MRI data as mentioned previously.

Table 2 presents the calculated pass rates, by 3-D Gamma analysis, achieved within regions determined by the isodose lines of different percentages of the TPS-calculated maximum dose of 8 Gy in the axial, coronal and sagittal central slices. The results showed that 3-D gamma achieved significantly higher pass rates than 2-D Gamma analysis, where pass rates of more than 90% were achieved for all regions in all cross sections, with the sagittal plane showing almost perfect agreement. The results confirmed that the dose distributions derived from R2-MRI maps closely match those calculated from the TPS, especially in the high dose regions, providing a promising accurate verification tool for dose delivery in radiotherapy. On the other hand, 3D Gamma analysis has shown lower pass rates when performed using 2%/2 mm criteria. The 3D Gamma analysis achieved pass rates of 96%, 82%, and 95% for the axial, coronal, and sagittal cross sections, respectively within the region at 90% of the maximum dose; while the pass rates within the regions at 70% and 50% of the maximum dose ranged between 72% and 90% for all cross sections. However, a more thorough investigation of Gamma analysis is still needed in future using tighter thresholds (2%/2 mm and 1%/1 mm) to further characterize and improve the spatial resolution and small-field performance of the ACAGLPVA gel dosimeter for CyberKnife applications.

**Table 2:** 3-D Gamma analysis (3%/ 3 mm) pass rates calculated within regions at different percentages of the TPS-calculated maximum dose of 8 Gy for the axial, coronal and sagittal central slices.

Percentage of max. dose in TPS data	Axial slice pass rate	Cor. slice pass rate	Sag. slice pass rate
90%	99.3%	92.8%	100%
70%	90.6%	93.8%	99.9%
50%	93.6%	95.6%	99.6%
20%	95.2%	–	–

### 3.4. Discussion

The performance of the proposed ACAGLPVA hydrogel dosimeter can be placed within the context of previously reported three-dimensional gel dosimetry systems used for radiotherapy verification. Polymer and hydrogel dosimeters have been evaluated for volumetric dose verification using optical CT, X-ray CT, cone-beam CT and MRI-based readout, with reported spatial accuracy and dosimetric agreement strongly dependent on gel formulation and imaging modality [12,20,21,24–26]. Recent CT-based studies, such as the optimized PASSAG polymer gel reported by Lumley et al. [29], demonstrated high sensitivity and linear dose response over clinically relevant dose ranges, supporting the feasibility of CT-read gel dosimetry for three-dimensional verification. Similarly, Kozicki et al. [30] employed multiple polymer gel formulations in combination with iterative cone-beam CT and dedicated 3D analysis software for routine radiotherapy quality assurance, highlighting the practical implementation of volumetric gel dosimetry in clinical workflows.

MRI-based gel dosimetry offers complementary advantages, including high soft-tissue contrast and voxel-wise mapping of intrinsic magnetic parameters without additional ionizing radiation during readout. In comparison with previously reported MRI-based polymer gel studies, the present work demonstrates comparable or higher gel dosimeter’s sensitivity for a stereotactic CyberKnife delivery, despite the stringent spatial gradients associated with small-field radiosurgery [36]. These results indicate that the glucose-sensitized ACAGLPVA gel provides volumetric dose verification performance consistent with established three-dimensional gel dosimeters, while offering a formulation specifically optimized for MRI-based R2 mapping. A key strength of this work is the use of a clinically derived CyberKnife patient-specific QA plan, providing a realistic and challenging small-field stereotactic delivery with steep dose gradients. The high gamma pass rates in high-dose regions suggest that MRI-based R2 mapping of the ACAGLPVA hydrogel can capture clinically relevant three-dimensional dose distributions for stereotactic quality assurance. In addition, MRI readout enables non-destructive volumetric assessment without additional ionizing radiation, supporting its potential as a complementary end-to-end verification tool in highly conformal radiotherapy workflows. The minor spatial differences between TPS-calculated and R2-MRI–derived dose distributions are likely influenced by factors inherent to MRI-based gel dosimetry and multimodality registration, including image noise, magnetic field inhomogeneity, uncertainty in voxel-wise mono-exponential R2 fitting, partial-volume effects in steep-gradient regions and residual CT–TPS–MRI registration errors. Limited MRI field of view in the slice direction and indirect dose derivation from R2 mapping may further contribute to localized deviations, particularly in lower-dose regions.

Beyond external beam radiotherapy quality assurance, the use of non-toxic, MRI-compatible hydrogel dosimeters also presents potential translational opportunities in clinical scenarios where true three-dimensional dosimetry remains challenging. Applications such as intraoperative radiotherapy (IORT) and intracavitary brachytherapy (IBT) are characterized by irregular target geometries, rapidly varying dose gradients and limited accessibility for conventional dosimetric tools [37,38]. In these settings, volumetric gel dosimeters could provide unique advantages by conforming to complex cavities and enabling post-irradiation three-dimensional dose assessment using MRI-based readout. Recent clinical studies in IORT and IBT have highlighted the need for improved verification methodologies capable of capturing spatial dose heterogeneity in confined anatomical regions [39,40]. The non-toxic nature and MRI compatibility of glucose-sensitized hydrogels, such as the ACAGLPVA formulation investigated in this study, support their conceptual applicability to these emerging use cases, although dedicated investigations are required to validate performance under clinically specific irradiation and geometric conditions.

In acrylic acid–based PVA hydrogel dosimeters, inorganic metal salts such as MgCl₂ have previously been employed as sensitizers to enhance radiation-induced polymerization and increase dose sensitivity [41,42]. While such formulations can yield relatively steep dose–response slopes, they may introduce practical limitations related to toxicity, optical clarity and susceptibility-related artifacts in MRI. In the present work, organic glucose was used as an alternative sensitizer to improve safety and compatibility with MRI-based readout. The measured R2–dose calibration for the glucose-sensitized ACAGLPVA gel demonstrates a strong linear response over the investigated dose range, confirming adequate sensitivity for stereotactic radiotherapy quality assurance. Although the absolute sensitivity (0.20 s^-1^Gy^-1^) may be moderately lower than that reported for some metal-salt–sensitized formulations (0.27 s^-1^Gy^-1^) [32], the achieved signal-to-noise ratio and high gamma pass rates indicate that this sensitivity is sufficient for accurate three-dimensional dose verification in high-dose regions. These observations support glucose sensitization as a deliberate design trade-off that favors improved safety, clarity and MRI compatibility while maintaining clinically relevant dosimetric performance.

The present study represents a feasibility evaluation of MRI-based R2 mapping for three-dimensional dose verification using the ACAGLPVA hydrogel dosimeter and several limitations should be acknowledged. It should be noted that, for the small spherical targets investigated in this study (7 mm diameter), a 3 mm distance-to-agreement represents a relatively coarse spatial tolerance. Consequently, the reported gamma pass rates may overestimate agreement in regions with very steep dose gradients. Although preliminary results of 3D Gamma analysis using 2%/2 mm criteria were promosing, the use of more stringent criteria (e.g., 2%/2 mm or 1%/1 mm) would provide increased sensitivity to spatial deviations and is planned as part of future work aimed at defining the ultimate spatial resolution limits of the ACAGLPVA gel dosimeter for CyberKnife applications. The experimental validation was based on a single CyberKnife patient-specific quality assurance plan with a maximum dose of 8 Gy. Although this case is representative of stereotactic radiosurgery, broader generalizability will require validation across multiple treatment plans, target sizes and dose prescriptions. In addition, the limited MRI field of view in the slice direction restricted assessment of low-dose regions in the coronal and sagittal planes, thereby confining gamma analysis in these views to higher isodose levels.Additional sources of uncertainty include image noise, fitting uncertainty in the mono-exponential R2 model, partial-volume effects in steep dose-gradient regions and residual registration errors between CT, TPS and MRI datasets. Potential influences of gel temperature, timing between irradiation and MRI acquisition, scan-to-scan repeatability and batch-to-batch formulation variability were not systematically evaluated in this study and may affect absolute R2 values; however, these factors are not expected to significantly impact the relative spatial dose comparison presented here. Furthermore, factors such as gel temperature stability, timing between irradiation and MRI scanning and batch-to-batch formulation variability were not systematically evaluated and may affect reproducibility. Future work will focus on repeatability studies, uncertainty quantification and expanded multi-plan validation to further assess the robustness and clinical applicability of the proposed approach.

## 4. Conclusions

This study has examined the effectiveness and accuracy of using the R2-MRI maps of the Acrylic acid with glucose PVA (ACAGLPVA) hydrogel dosimeter as a reliable tool for the 3-D verifification of radiation doses. The results showed high agreement when comparing the dose distribution maps calculated from the TPS to those derived from the R2-MRI maps of the (ACAGLPVA) hydrogel phantom as evident from both 2-D and 3-D Gamma analysis. The pass rates achieved by the 3-D Gamma analysis with 3% dose difference and 3-mm distance-to-agreement criteria consistently exceeded 90% for all planes when evaluated within isodose contours down to 20% of the maximum dose for axial plane and to 50% for sagittal and coronal planes. The results of this study support the potential use of R2-MRI-based dosimetry of the (ACAGLPVA) gel for 3-D verification of complex radiotherapy plans. Future research may focus on optimizing the gel composition and MRI protocols to further refine dosimetric accuracy and broaden clinical applicability.
