# Correction: Evaluation of an acrylic acid hydrogel dosimeter for 3-D dose verification in radiotherapy using MRI

**DOI:** 10.1371/journal.pone.0350778

**Published:** 2026-06-01

**Authors:** Ihssan S. Masad, Khalid A. Rabaeh, Samer I. Awad, Akram A. Almousa, Ahmed M. Masawi, M. Abdullah Al Kafi, Samer M. Alheet, Abdallah J. Nofal, Belal Moftah

The following information is missing from the Funding statement: APC was supported by Gulf University for Science and Technology (GUST), Kuwait.

## References

[1] Masad IS, Rabaeh KA, Awad SI, Almousa AA, Masawi AM, Al Kafi MA, et al. Evaluation of an acrylic acid hydrogel dosimeter for 3-D dose verification in radiotherapy using MRI. PLoS One. 2026;21(5):e0349404. doi: 10.1371/journal.pone.0349404 42133669 PMC13175346

